# Adenylyl cyclase-5 in the dorsal striatum function as a molecular switch for the generation of behavioral preferences for cue-directed food choices

**DOI:** 10.1186/s13041-014-0077-7

**Published:** 2014-11-07

**Authors:** Hannah Kim, Tae-Kyung Kim, Ji-Eun Kim, Jin-Young Park, Yunjin Lee, Minkyung Kang, Kyoung-Shim Kim, Pyung-Lim Han

**Affiliations:** Department of Brain and Cognitive Sciences, Ewha Womans University, 11-1 Daehyun-Dong, Seodaemoon-Gu, Seoul 120-750 Republic of Korea; Laboratory Animal Center, Korea Research Institute of Bioscience and Biotechnology, Daejeon, Republic of Korea; Brain Disease Research Institute, Ewha Womans University, 11-1 Daehyun-Dong, Seodaemoon-Gu, Seoul 120-750 Republic of Korea; Department of Chemistry and Nano Science, Ewha Womans University, 11-1 Daehyun-Dong, Seodaemoon-Gu, Seoul 120-750 Republic of Korea

**Keywords:** AC5, mGluRs, Preferences, Choices, Dorsal striatum, Sensory integration

## Abstract

**Background:**

Behavioral choices in habits and innate behaviors occur automatically in the absence of conscious selection. These behaviors are not easily modified by learning. Similar types of behaviors also occur in various mental illnesses including drug addiction, obsessive-compulsive disorder, schizophrenia, and autism. However, underlying mechanisms are not clearly understood. In the present study, we investigated the molecular mechanisms regulating unconditioned preferred behaviors in food-choices.

**Results:**

Mice lacking adenylyl cyclase-5 (AC5 KO mice), which is preferentially expressed in the dorsal striatum, consumed food pellets nearly one after another in cages. AC5 KO mice showed aversive behaviors to bitter tasting quinine, but they compulsively chose quinine-containing AC5 KO-pellets over fresh pellets. The unusual food-choice behaviors in AC5 KO mice were due to the gain of behavioral preferences for food pellets containing an olfactory cue, which wild-type mice normally ignored. Such food-choice behaviors in AC5 KO mice disappeared when whiskers were trimmed. Conversely, whisker trimming in wildtype mice induced behavioral preferences for AC5 KO food pellets, indicating that preferred food-choices were not learned through prior experience. Both AC5 KO mice and wildtype mice with trimmed whiskers had increased glutamatergic input from the barrel cortex into the dorsal striatum, resulting in an increase in the mGluR1-dependent signaling cascade. The siRNA-mediated inhibition of mGluR1 in the dorsal striatum in AC5 KO mice and wildtype mice with trimmed whiskers abolished preferred choices for AC5 KO food pellets, whereas siRNA-mediated inhibition of mGluR3 glutamate receptors in the dorsal striatum in wildtype mice induced behavioral preferences for AC5 KO food pellets, thus mimicking AC5 KO phenotypes.

**Conclusions:**

Our results show that the gain and loss of behavioral preferences for a specific cue-directed option were regulated by specific cellular factors in the dorsal striatum, such that the preferred food choices were switched on when either the mGluR3-AC5 pathway was inactive or the mGluR1 pathway was active, whereas the preferred food-choices were switched off when mGluR1 or its downstream pathway was suppressed. These results identify the AC5 and mGluR system in the dorsal striatum as molecular on/off switches to direct decisions on behavioral preferences for cue-oriented options.

## Background

Behavioral decisions in goal-directed actions are consciously produced through learned and expected outcome values, whereas habits are initially learned and are expressed on the basis of acquired action values [1,2]. In contrast, innate behaviors form without being based on previous experience, but are produced on the basis of programmed action values [3]. Behavioral choices in habits and innate behaviors are produced automatically in the absence of conscious selection. These behaviors are not easily modified by learning. Similar types of behaviors also occur in various mental illnesses including drug addiction, obsessive-compulsive disorder, schizophrenia, and autism [4,5]. However, underlying mechanisms are not clearly understood and treatment strategies need to be developed.

Neuroimaging studies of humans [6,7], recording and behavior studies of monkeys [8,9] and rodents [10,11], and lesion and pharmacological studies of rats [12,13] have reported the importance of the medial prefrontal cortex and dorsal striatum in various goal-oriented tasks and habits. The medial prefrontal cortex including the prelimbic and infralimbic cortices in rodents projects to the dorsomedial striatum (the caudate in humans) [14,15], and supports goal-directed behaviors and behavioral flexibility [5,16]. This cortico-strital cognitive loop regulates decisions in various stimulus–response (SR) learning tasks [17]. Whereas the sensorimotor cortex projects to the dorsolateral striatum (the putamen in humans) [2,15], and this cortico-strital loop is involved in decisions in SR habit formation [6,18]. The dorsomedial striatum is selectively activated during initial learning and its activity decreases with extended training, whereas the dorsolateral striatum becomes more active after the automaticity of a habit develops [19]. Innate behaviors are not learned, but genetically programmed in the nervous system [3].

The dorsal striatum receives glutamatergic inputs from various parts of the cerebral cortex including the associative cortex, the limbic cortex, and the sensory motor cortex, which are composed of associative/cognitive, limbic, and sensory motor corticostriatal loops, respectively [20-22]. Functional correlates of the corticostriatal circuits suggest that different corticostriatal glutamatergic inputs and their receptive neural components in the dorsal stratum support various decision making processes. However, little is known about how sensory information is integrated, how a particular option is selected, and how final specific behavioral outputs are produced.

Adenylyl cyclase type 5 (AC5) is preferentially expressed in the dorsal striatum and nucleus accumbens along with broad expression, to a lesser extent, in the other brain regions including the prefrontal cortex [23,24]. Owing to its essential function as an effector for multiple receptors and as a regulator for cAMP-signaling signaling cascades in various brain regions, AC5 KO mice are resistant to the haloperidol actions [23], morphine actions [25], ethanol effects [26,27], and L-DOPA-induced dyskinesia [28], and show reduced anxiety [29] and reduced pain responses [30], but they are highly sensitive to stress [31]. Thus, AC5 is a key component in the striatum and prefrontal cortex, and so functions as a gate-keeper in various cognitive and emotional behaviors.

The present study dissects the neural and molecular substrates mediating behavioral choice for an olfactory cue-driven option by targeting specific genes expressed in the corticostriatal circuit using genetic, pharmacological, and molecular tools.

## Results

### AC5 KO mice showed preferred choices for AC5 KO food pellets over WT food pellets

**Figure 1:**
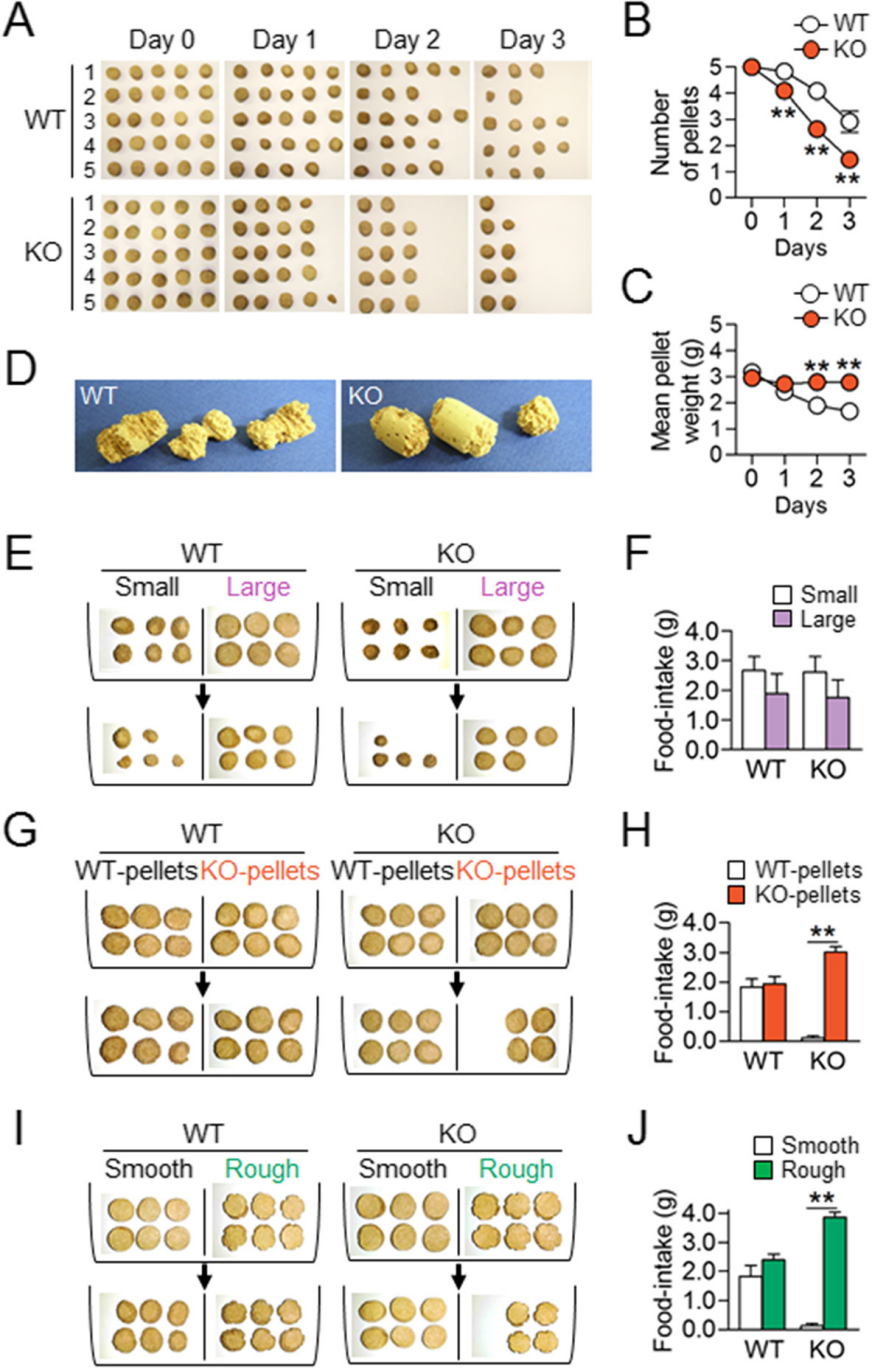
**AC5 KO mice gained preferred behaviors for specific cue-directed options. (A-C)** Photographs showing representative sets of food pellets consumed by 5 individuals of WT mice (WT) and AC5 KO mice (KO) for 3 days **(A)**. Initially, five fresh food pellets were presented to each mouse and the mean number **(B)** and weight **(C)** of food pellets remaining on the next day were recorded. Two-way repeated measures ANOVA, Holm-Sidak post hoc test: for pellet numbers, genotype [*F*_(1,20)_ = 17.91, *p* < 0.001], time [*F*_(3,60)_ = 138.7, *p* < 0.001], and genotype × time interaction [*F*_(3,60)_ = 6.935, *p* < 0.001]; for pellet weight, genotype [*F*_(1,20)_ = 29.24, *p* < 0.001], time [*F*_(3,60)_ = 43.13, *p* < 0.001], and genotype × time interaction [*F*_(3,60)_ = 29.49, *p* < 0.001]. **(D)** Photographs showing cork rods chewed by WT and AC5 KO mice. Three cork rods were presented to each mouse in the absence of food pellets and the cork rods remaining on the next day were collected and photographed. **(E, F)** Photographs showing food-intake for small vs. large food pellets by WT and AC5 KO mice **(E)** and its quantification **(F)**. Three large (1.5-2.0 cm in diameter) and three small (0.5-1.0 cm in diameter) pellets were presented to each mouse and food pellets remaining on the next day were recorded. **(G-J)** KO mice preferred KO food pellets to WT pellets **(G, H)** and rough-pellets to smooth- pellets **(I, J)**. Three WT pellets *vs.* three KO pellets **(G)** or three rough *vs.* three smooth pellets **(I)** were presented to each mouse and food pellets remaining on the next day were recorded **(H, J)**. Two-way ANOVA and *Tukey's* HSD test: for both **(H, J)**, no genotype effect, significant food effect, and significant genotype × food interaction. Data are presented as the mean ± SEM (n = 8-11). * and ** denote the difference between indicated groups at *p* <0.05 and *p* <0.01.

AC5 KO mice exhibited unusual food-intake behaviors, tending to finish one food pellet to the end before starting to eat the next in their home cages, whereas wildtype (WT) mice consumed food pellets near-randomly (Figure 1A-C). When presented with cork rods in a food-pellet size (1.5 cm in diameter × 2.5 cm in length), AC5 KO mice somewhat obsessively nibbled the one that they had been taking, whereas WT mice chewed them indiscriminately (Figure 1D). However, AC5 KO mice did not seek small food pellets over large ones (Figure 1E and F). When simultaneously presented with food pellets that wildtype mice had been taking (WT pellets) and size-matched food pellets that AC5 KO mice had been taking (KO pellets), AC5 KO mice greatly preferred KO pellets over WT pellets (Figure 1G and H). AC5 KO mice showed another preference for food pellets with undulating surfaces (rough pellets) over food pellets with smooth surfaces (smooth pellets) (Figure 1I and J). These results suggest that AC5 KO mice displayed altered food-choice behaviors based on certain sensory cues.

### AC5 KO mice differentiated KO food pellets based on an olfactory cue produced by AC5 KO mice

**Figure 2:**
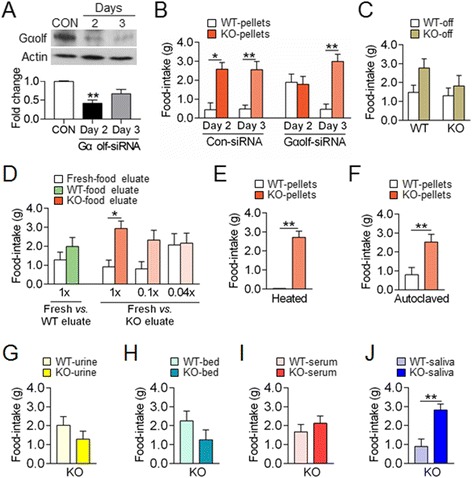
**AC5 KO mice produced behavioral preferences for KO pellets based on an olfactory cue-directed option. (A, B)** Western blots showing the siRNA-mediated down-regulation of the Gαolf levels in the olfactory epithelium in WT mice and their quantification **(A)**. Blocking of the preferred choices for KO food pellets in AC5 KO mice after the infusion of Gαolf-siRNA into the olfactory epithelium **(B, C)** Behavioral choices of AC5 KO mice for KO pellets *vs.* WT pellets whose surfaces were peeled off. **(D)** Behavioral choices of AC5 KO mice for fresh pellet pasted with KO-food eluate *vs.* fresh-food eluate at gradually diluted concentrations and for fresh pellet pasted with WT-food eluate *vs.* fresh-food eluate. **(E, F)** Behavioral choices of AC5 KO mice for WT pellets *vs.* KO pellets that were heated **(E)** or autoclaved **(F). (G-J)** Behavioral choices of AC5 KO mice for food pellets that were pasted with WT urine *vs.* KO urine **(G)**, for food pellets that were mingled with WT bed *vs.* KO bed **(H)**, for food pellets that were pasted with WT serum *vs.* KO serum **(I)**, and for food pellets that were pasted with WT saliva *vs.* KO saliva **(J)**. Data were mean ± SEM (n = 7-12). * and ** denote the difference between indicated groups at p <0.05 and p <0.01, respectively. One-way ANOVA, Tukey's HSD post hoc test **(A)**, Two-way repeated measures ANOVA, Holm-Sidak post hoc test **(B)**, Two-way ANOVA and *Tukey's* HSD post hoc test **(C, D)**, and Student *t*-test **(D, E-J)** were used.

We examined whether or not behavioral preferences for KO pellets in AC5 KO mice relied upon the olfactory sensory system. The infusion of siRNA-Gαolf (the guanine nucleotide-binding protein subunit αolf, a key player in the initial olfaction step) into the olfactory epithelium of AC5 KO mice suppressed preferred choices for KO pellets over WT pellets, indicating that AC5 KO mice exhibited behavioral choice for KO pellets based on an olfactory cue (Figure 2A and B).

AC5 KO mice showed no preference for KO pellets whose surface was removed (Figure 2C). The surface of collected KO pellets was incubated in 4 volumes of water with moderate shaking for 20 min and after centrifugation the supernatant was collected. AC5 KO mice discriminated and displayed enhanced choice for the food pellets pasted with the KO pellets eluate over the pellets pasted with the fresh-food eluate, but this behavioral sensitivity disappeared with the dilution of the KO-pellet eluate (Figure 2D). In contrast, AC5 KO mice showed no preference for the food pellets pasted with the WT-food eluate (Figure 2D). These results indicate that the olfactory cue detected by AC5 KO mice was present on the surface of KO pellets and was extractable in water. AC5 KO mice discriminated between boiled and autoclaved KO pellets from the respective WT-pellet control (Figure 2E and F), indicating that the olfactory cue itself was heat resistant.

AC5 KO mice did not prefer food pellets pasted with KO urine (Figure 2G), those mingled with the KO cage bed (Figure 2H), or those pasted with KO blood serum (Figure 2I), but preferred food pellets pasted with KO saliva (Figure 2J).

### AC5 KO mice showed reduced, but significant levels of behavioral preferences for AC5 KO food pellets in the presence of quinine

**Figure 3:**
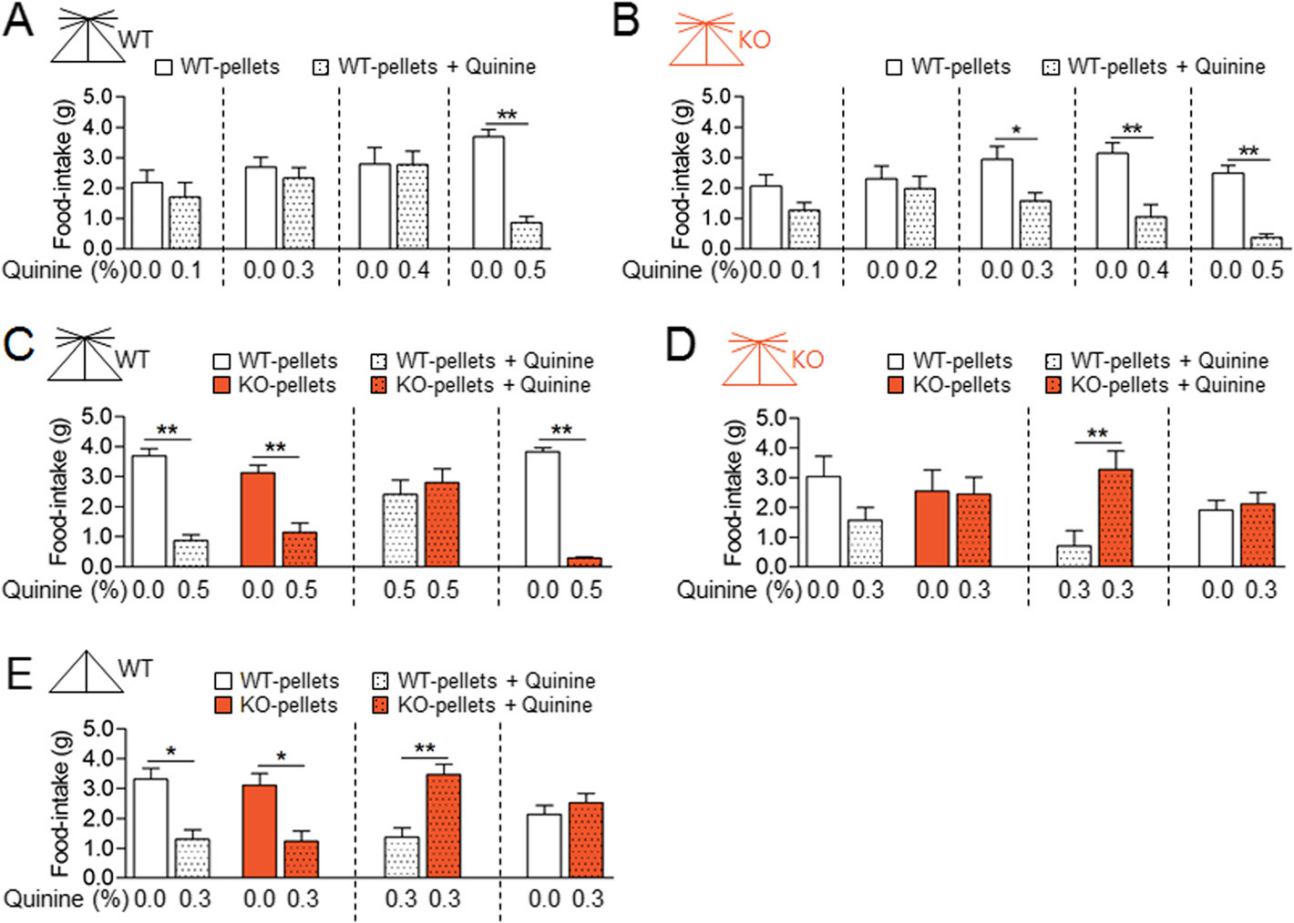
**AC5 KO mice moderately preferred AC5 KO food pellets in the presence of quinine. (A, B)** Behavioral preferences of WT mice and AC5 KO mice at a choice of WT pellets *vs.* WT pellets pasted with quinine at different doses. Two-way ANOVA and *Tukey’s* HSD test: for quinine-dose responses by WT mice **(A)**, quinine [*F*_(1,68)_ = 9.815, *p* = 0.0026], no food effect, and quinine x food interaction [*F*_(3,68)_ = 4.799, *p* = 0.0043]; for quinine-dose responses by KO mice **(B)**, quinine [*F*_(1,82)_ = 32.11, *p* = 0.001], no food effect [*F*_(4,82)_ = 1.833, *p* = 0.1304], and no quinine x food interaction. **(C, D)** Behavioral preferences of WT mice and AC5 KO mice at a choice of WT pellets *vs.* quinine-pasted WT pellets, KO pellets *vs*. quinine-pasted KO pellets, and WT pellets *vs.* quinine-pasted KO pellets. Two-way ANOVA and *Tukey’s* HSD test: for behavioral choices by WT mice **(C)**, quinine [*F*_(1,18)_ = 23.91, *p* <0.001], no food effect, and no quinine x food interaction; for behavioral choices by KO mice **(D)**, no quinine effect, no food effect, and no quinine × food interaction [*F*_(1,18)_ = 0.9741, *p* = 0.3376]. **(E)** Behavioral preferences of WT mice with trimmed whiskers at a choice of WT pellets *vs.* quinine-pasted WT pellets, KO pellets *vs*. quinine-pasted KO pellets, and WT pellets *vs.* quinine-pasted KO pellets. Two-way ANOVA and *Tukey’s* HSD test: quinine [*F*_(1,13)_ = 18.47, *p* <0.001], no food effect [*F*_(1,13)_ =0.3199, *p* =0.9931], and quinine x food interaction [*F*_(1,13)_ = 0.0245, *p* = 0.8779]. Data are presented as the mean ± SEM (n = 6-12). * and ** denote the difference between indicated groups at *p* <0.05 and *p* <0.01.

A series of experiments were carried out to test whether behavioral preferences for AC5 KO food pellets in AC5 KO mice and in WT mice with cut whiskers occurred compulsively. Both WT mice and AC5 KO mice showed aversive behaviors to WT pellets whose surfaces were pasted with 0.5% of the bitter substance quinine, but AC5 KO mice expressed aversive responses to starting from 0.3% of quinine, thus showing slightly more sensitive to quinine than WT mice (Figure 3A and B).

WT mice showed aversive behaviors to 0.5% of quinine regardless of being presented on WT pellet or KO pellets and preferentially chose food pellets that carried no quinine over quinine-pasted pellets (Figure 3C). AC5 KO mice tended to show aversive responses to 0.3% of quinine, but they significantly preferred quinine-KO pellets at a choice of quinine-KO pellets *vs.* quinine-WT pellets, whereas at a choice of KO pellets vs. quinine-KO pellets, and WT pellets vs. quinine-KO pellets, they chose both (Figure 3D). WT mice with cut whiskers showed aversive responses to 0.3% of quinine, but they also preferred quinine-KO pellets at a choice of quinine-KO pellets *vs.* quinine-WT pellets (Figure 3E).

### Whisker trimming in wildtype mice and AC5 KO mice switched on or off the behavioral choices for a cue-directed option in opposite ways

**Figure 4:**
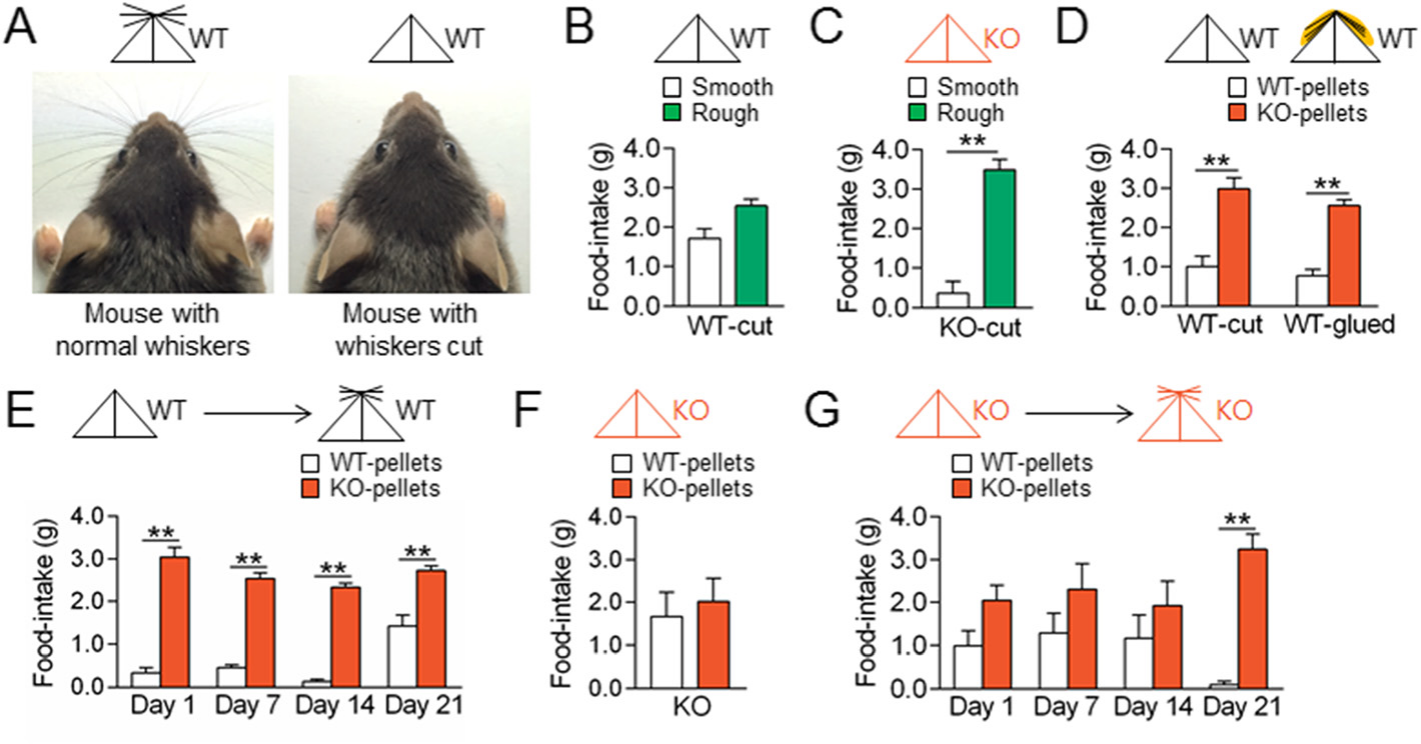
**Whisker trimming in WT and AC5 KO mice switched on-and-off behavioral preferences for KO food pellets in opposite ways. (A)** Photographs showing a mouse with normal whiskers or whiskers cut to the fur level and respective mouse symbols. **(B, C)** Whisker trimming in WT **(B)** and AC5 KO mice **(C)** had no effect on their behavioral preferences for rough pellets over smooth ones. **(D)** Whisker trimming or whiskers glued to the snout and chin in WT mice induced behavioral preferences for KO pellets over WT pellets. **(E)** Temporal changes of whisker trimming effects in WT mice on behavioral preferences for KO pellets over WT pellets. Two-way repeated measures ANOVA, Holm-Sidak post-hoc test: time [*F*_(3, 42)_ = 11.22, *p* <0.001], food [*F*_(1,14)_ = 423.4, *p* <0.001], and time × food interaction [*F*_(3,42)_ = 7.517, *p* <0.001]. **(F)** Whisker trimming effects in AC5 KO mice on behavioral preferences for KO pellets over WT pellets. **(G)** Temporal changes of whisker trimming effects in AC5 KO mice on behavioral preferences for KO pellets over WT pellets. Two-way repeated measures ANOVA, Holm-Sidak test: time [*F*_(3, 36)_ = 0.7297, *p* = 0.5410], food [*F*_(1,12)_ = 25.95, *p* <0.001], and time x food interaction [*F*_(3,36)_ = 2.832, *p* = 0.0519]. Mouse symbols: WT (black) and AC5 KO (red) with or without whiskers. Data are presented as the mean ± SEM (n = 7-20), * and ** denote the difference between indicated groups at *p* <0.05 and *p* <0.01.

A potential role of whiskers in the discrimination of rough food pellets was examined. WT mice with the vibrissa cut to the fur level (Figure 4A) showed similar behavioral preferences as those of WT mice with intact whiskers for rough pellets over smooth ones (Figure 4B). AC5 KO mice with cut whiskers also retained the same behavioral preference for rough pellets over smooth ones (Figure 4C) as AC5 KO mice with intact whiskers (Figure 1I and J).

Unexpectedly however, WT mice with cut whiskers or WT mice with intact whiskers that were glued onto the snout and chin both exhibited behavioral preferences for KO pellets over WT pellets (Figure 4D). With the growth of whiskers, the newly acquired preference for KO pellets slowly diminished, although the effect of whisker trimming in WT mice moderately remained on day 21 (Figure 4E). On the other hand, whisker trimming in AC5 KO mice abolished behavioral preference for KO pellets over WT ones (Figure 4F). However, AC5 KO mice completely re-gained behavioral preference for KO pellets on day 21 as whiskers regrew (Figure 4G). These results indicate that behavioral preferences for KO pellets displayed by AC5 KO mice should not be attributed to developmental changes, but they were modified by a neuronal activity change in the whisker sensory pathway, and thus, were not learned through prior experience.

### The selective suppression of AC5 in the dorsal striatum replicated the behavioral choice of KO food and rough food pellets

**Figure 5:**
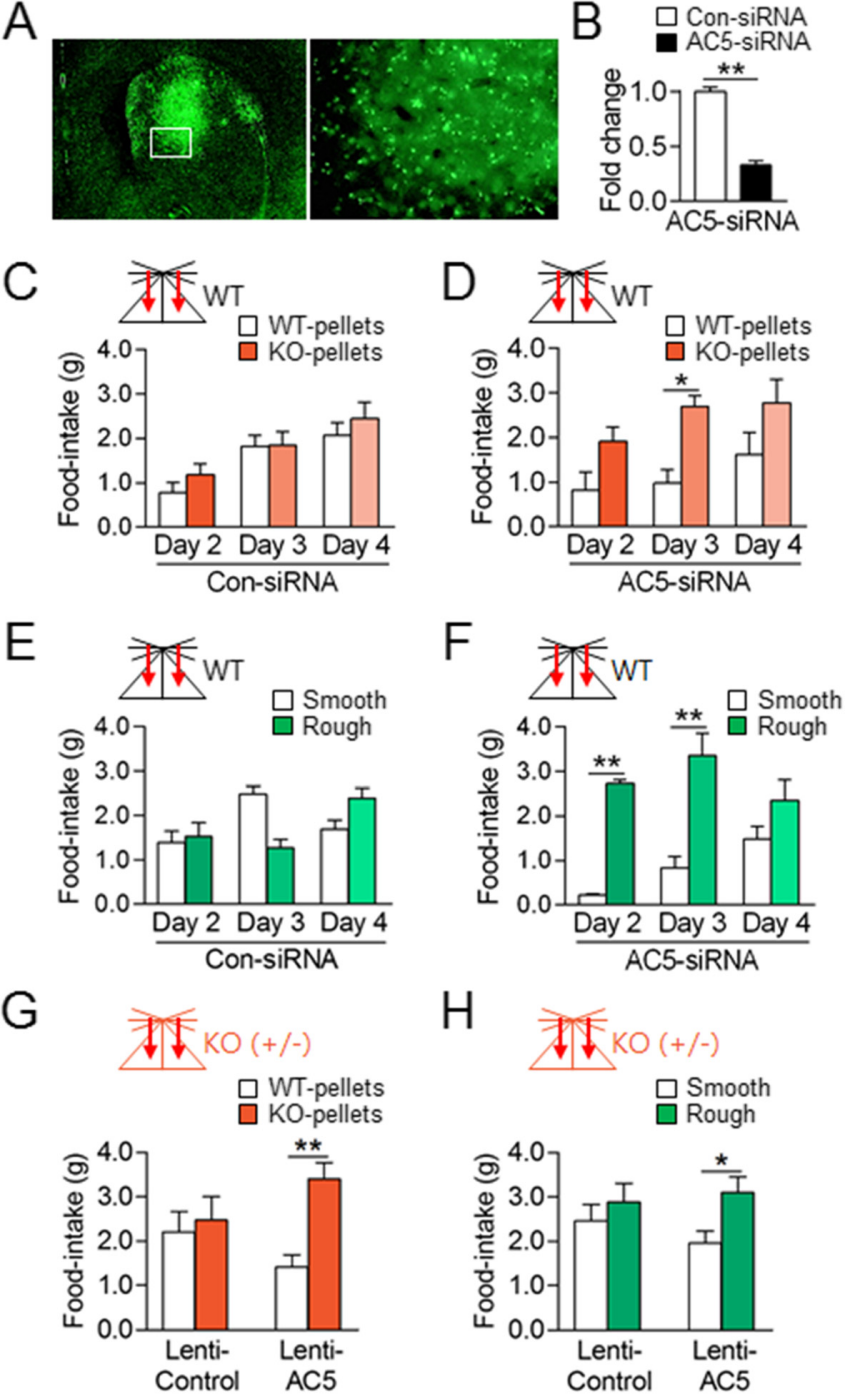
**The suppression of AC5 in the dorsal striatum replicated behavioral preferences for KO food pellets and rough pellets. (A, B)** Photomicrographs showing the dorsal striatum injected with siGLO control and high magnification of the rectangle **(A)**. Real-time PCR showing siRNA-mediated knockdown of AC5 in the dorsal striatum **(B)**. **(C-F)** The siRNA-mediated knockdown of AC5 in the dorsal striatum in WT mice induced behavioral preferences for KO pellets over WT pellets **(C, D)** and rough pellets over smooth pellets **(E, F)**; the siRNA effects in both cases disappeared on day 4. Two-way repeated measures ANOVA, Holm-Sidak test: for Con-siRNA **(C)**, time [*F*_(2, 44)_ = 13.25, *p* <0.001], no food effect, and no interaction; for AC5-siRNA **(D)**, no time effect, food [*F*_(1, 18)_ = 15.73, *p* <0.001], and no interaction; for Con-siRNA **(E)**, no time effect, no food effect, and significant interaction [*F*_(2, 28)_ = 8.507, *p* = 0.0013]; for AC5-siRNA (F), time [*F*_(2, 20)_ = 3.635, *p* = 0.0450], food [*F*_(1, 10)_ = 19.89, *p* = 0.0012], and no interaction. **(G, H)** Lenti-AC5-shRNA-mediated knockdown of AC5 in the dorsal striatum in heterozygote AC5 KO mice (AC5^+/−^) mice induced preferred choices for AC5 KO-pellets over WT-pellets **(G)** and rough pellets over smooth pellets **(H)**. Food-choice tests were started from 10 days after the Lenti-injection. Two-way ANOVA and *Tukey’s* HSD test: for Lenti-AC5-shRNA effects on KO pellets **(G)**, food [*F*_(1,60)_ = 7.916, *p* = 0.007], no AC5-shRNA effect, and significant interaction [*F*_(1,60)_ = 4.625 and *p* = 0.036]; for Lenti-AC5-shRNA effects on rough pellets **(H)**, food [*F*_(1,46)_ =  6.209, *p* = 0.0164], no AC5-shRNA effect, and no interaction. Mouse symbols: WT mice (black) with stereotaxic injections (arrows). Data were mean ± SEM (n = 11-19). * and ** denote difference between indicated groups at *p* <0.05 and *p* <0.01.

Concerned that the AC5 deficiency in AC5 KO mice affects the whole brain, the role of AC5 in the dorsal striatum in the expression of behavioral preferences for KO food pellets was examined. The siRNA-mediated suppression of AC5 in the dorsal striatum of WT mice induced behavioral preferences for KO pellets over WT ones and for rough pellets over smooth ones (Figure 5A-F). The infusion of Lenti-AC5-shRNA into the dorsal striatum, but not the nucleus accumbens (NAc), also induced behavioral preferences for KO pellets over WT ones and for rough pellets over smooth ones (Figure 5G and H). Injections of control-siRNA or Lenti-GFP control did not produce such behaviors. These results suggest that the selective depletion of AC5 in the dorsal striatum was sufficient to induce behavioral preferences for an olfactory- or tactile-cue dri-ven option.

### The gating of preferred food-choices was directed by an enhanced **glutamatergic** input into the dorsal striatum

**Figure 6:**
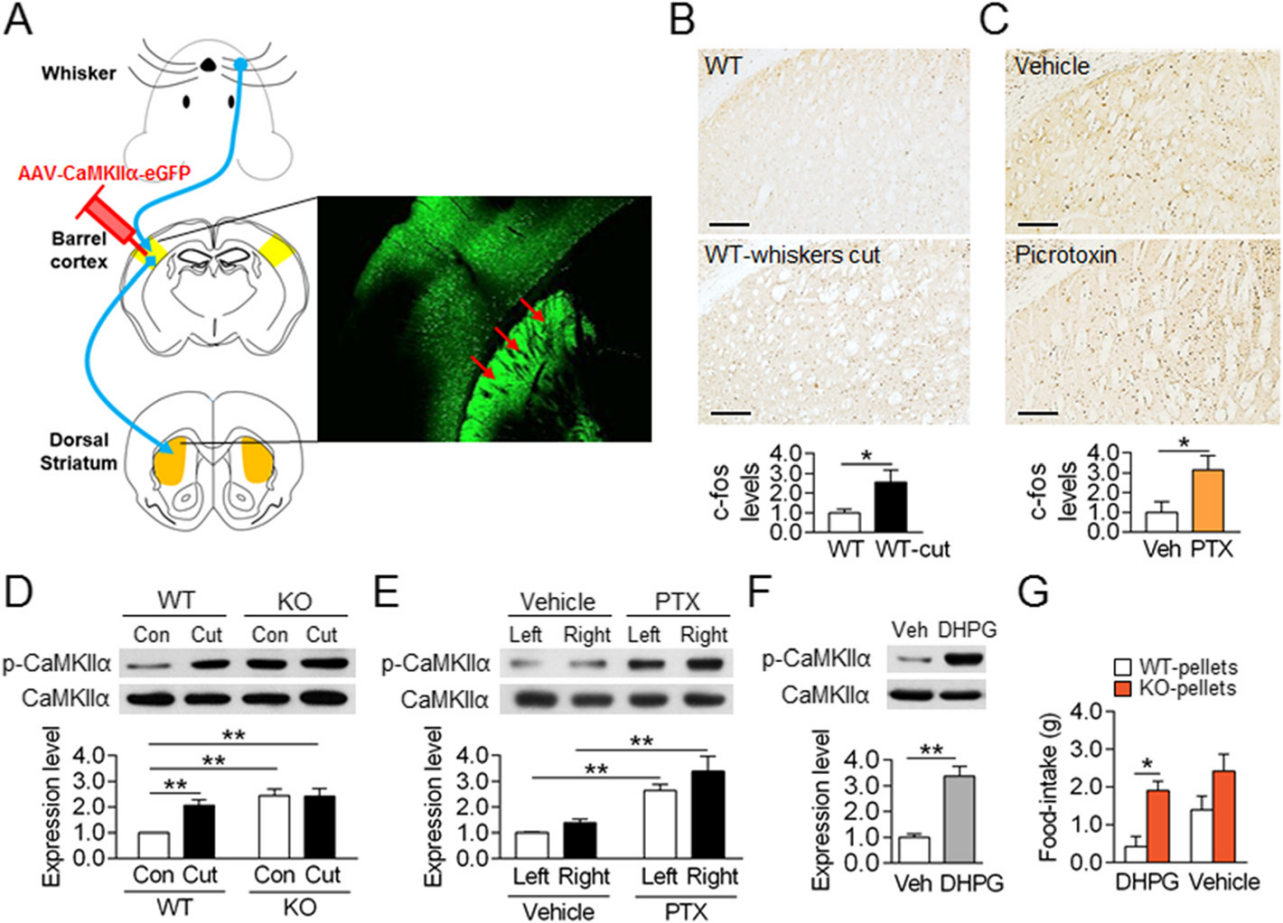
**Whisker trimming increased the activity of the glutamatergic input from the barrel cortex into the dorsal striatum. (A)** Schematic diagram showing the whisker sensory pathway heading, through the thalamus, to the barrel cortex, and the following corticostriatal projection. The corticostriatal projection was visualized by the injection of AAV2-CaMKIIα-eGFP into the barrel cortex. **(B, C)** Whisker trimming increased c-Fos induction in the dorsal striatum **(B)**. Examined 24 h after whisker trimming. The infusion of picrotoxin in the barrel cortex increased c-Fos induction in the dorsal striatum in 1 h **(C)**. Scale bar; 200 μm. **(D, E)** Whisker trimming in WT mice and the AC5 KO itself increased p-CaMKIIα in the dorsal striatum. p-CaMKIIα level was examined 24 h after whisker trimming **(D)**. Infusion of picrotoxin in the barrel cortex increased the p-CaMKIIα level in the dorsal striatum in 1 h **(E)**. Two-way ANOVA, Tukey’s HSD post hoc test; for whisker trimming **(D)**, whiskers-cut [*F*_(1, 28)_ = 7.243, *p* =0.0176], genotype [*F*_(1, 28)_ = 24.43, *p* <0.001], and whiskers-cut × genotype interaction [*F*_(1, 28)_ = 6.843, *p* = 0.0203]; for picrotoxin treatment **(E)**, picrotoxin [*F*_(1, 20)_ = 82.17, *p* <0.001], left-right [*F*_(1, 20)_ = 7.830, *p* = 0.0111], and no picrotoxin × left-right interaction. **(F)** Stereotaxic injection of DHPG into the dorsal striatum increased the p-CaMKIIα level in 30 min. **(G)** Infusion of DHPG into the dorsal striatum through the pre-implanted cannulas in WT mice induced behavioral preferences for AC5 KO pellets over WT pellets. Two-way ANOVA, Tukey’s HSD test; DHPG [*F*_(1, 24)_ = 4.520, *p* = 0.0440], food [*F*_(1, 24)_ = 13.38, *p* = 0.0012], but no DHPG × food interaction. Data are presented as the mean ± SEM (n = 5-8). * and ** denote the difference between indicated groups at *p* <0.05 and *p* <0.01.

Given the decisive role of the AC5 KO and whisker trimming in the expression of preferred food-choices, we examined whether the AC5 null and whisker trimming produce a common biochemical change in the dorsal striatum. In rodents, whisker sensory afferents are projected through the thalamus to layer 4 of the somatosensory cortex, called the barrel cortex [32]. The barrel cortex consists of glutamatergic projection neurons and GABAergic interneurons, and in this region, GABAergic neurons regulate the excitatory tone of glutamatergic projection neurons [33]. The injection of AAV-CaMKIIα-eGFP into the barrel cortex helped to visualize the corticostrital projection to the dorsal striatum (Figure 6A). Whisker trimming in WT mice increased c-Fos induction in the dorsal striatum (Figure 6B). The infusion of picrotoxin, a GABA_A_ receptor antagonist, into the barrel cortex, which enhances the excitability of the glutamatergic projection neurons as a result of disinhibition [34], increased c-Fos induction in the dorsal striatum in 1 h (Figure 6C). These results verify the presence of a functional connectivity between whiskers and dorsal striatum. Furthermore, WT mice with cut whiskers, which showed preferred choices for AC5 KO food pellets, had increased p-CaMKIIα levels in the dorsal striatum. AC5 KO mice also showed enhanced p-CaMKIIα level in the dorsal striatum, although whisker trimming in AC5 KO mice did not further change p-CaMKIIα level (Figure 6D). The infusion of picrotoxin into the barrel cortex of WT mice, which resulted in enhanced c-Fos induction in the dorsal striatum (Figure 6C), increased p-CaMKIIα level in the dorsal striatum (Figure 6E).

The dorsal striatum expressed group I metabotropic glutamate receptors (mGluR1/5) and group II mGluRs (mGluR2/3) at high levels [35]. Group II mGluRs antagonistically suppressed the function of group I mGluRs [36]. The injection of the mGluR1/5 agonist, 3,5-dihydroxyphenylglycine (DHPG), in the dorsal striatum increased p-CaMKIIα level (Figure 6F), mimicking the signaling state in WT mice with cut whiskers or in AC5 KO mice (Figure 6D). Indeed, the infusion of DHPG though the pre-implanted cannula in the dorsal striatum in WT mice induced behavioral preferences for KO pellets, but this behavior diminished on the following day when the vehicle was infused (Figure 6G).

### The mGluR1 and mGluR3 systems in the dorsal striatum regulated behavioral preferences for a cue-directing option in opposite directions

**Figure 7:**
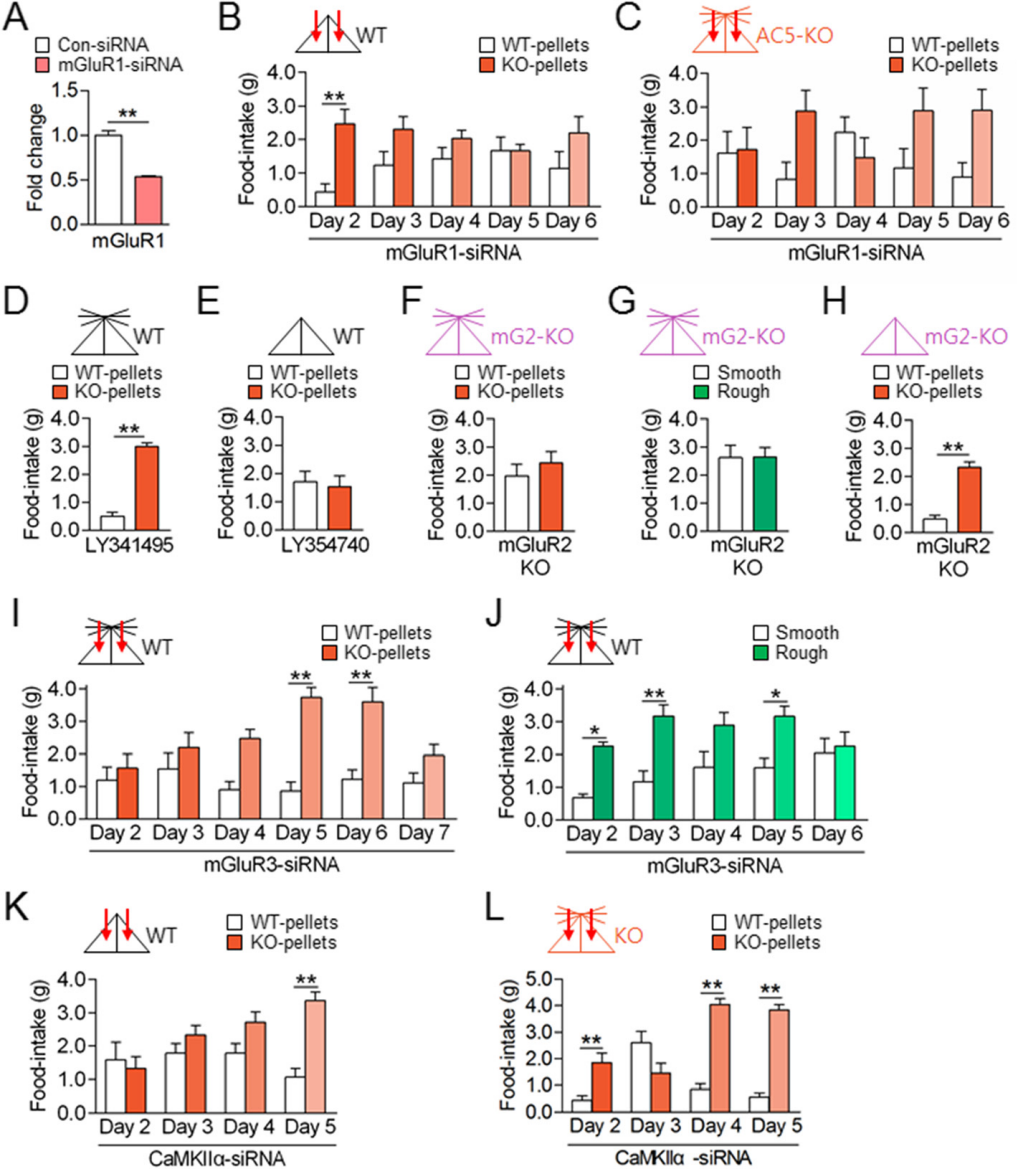
**The mGluR1 and mGluR3 signaling systems in the dorsal striatum antagonistically regulated behavioral preferences for AC5 KO food pellets. (A-C)** siRNA-mediated knockdown of mGluR1 in the dorsal striatum **(A)** suppressed behavioral preferences for AC5 KO pellets over WT pellets in WT mice with cut whiskers **(B)** and in AC5 KO mice **(C)**. Two-way repeated measures ANOVA, Holm-Sidak test: for mGluR1-siRNA **(B)**, food [*F*_(1, 12)_ = 11.15, *p* = 0.0059], no time effect and no interaction; for mGluR1-siRNA **(C)**, no food and no time effects, but food × time interaction [*F*_*(*4, 48)_ = 3.007, *p* = 0.0271]. **(D, E)** Administration of the mGluR2/3 antagonist LY341495 in WT mice induced behavioral preferences for KO food over WT food **(D)**. The mGluR2/3 agonist LY354740 in WT mice with cut whiskers blocked behavioral preferences for AC5 KO pellets **(E)**. **(F-H)** mGluR2 KO mice did not exhibit behavioral preferences for KO pellets over WT pellets **(F)** and rough food pellets **(G)**. Whisker trimming in mGluR2 KO induced behavioral preferences for KO pellets over WT pellets **(H)**. **(I, J)** Injection of mGluR3-siRNA in the dorsal striatum in WT mice induced behavioral preferences for KO pellets over WT pellets **(I)** and for rough pellets over smooth pellets **(J)**. Two-way repeated measures ANOVA, for mGluR3-siRNA **(I)**, significant food and time effects, and significant interaction; for mGluR3-siRNA **(J)**, significant food effect, but no time effect and no interaction. **(K, L)** Injection of CaMKIIα-siRNA into the dorsal striatum suppressed behavioral preferences for KO pellets over WT pellets in both WT mice with cut whiskers **(K)** and in AC5 KO mice **(L)**. Two-way repeated measures ANOVA for both **(K, L)**, significant food and time effects and significant interaction. Data are presented as the mean ± SEM (n = 7-9). * and ** denote the difference between indicated groups at *p* <0.05 and *p* <0.01.

Next, we examined which glutamate receptors in the dorsal striatum are involved in the production of preferred food-choice behaviors. The injection of mGluR1-siRNA in the dorsal striatum in WT mice with cut whiskers suppressed behavioral preferences for AC5 KO pellets over WT ones (Figure 7A and B), indicating that the effect of whisker trimming on the expression of preferred food-choice required mGluR1. The injection of mGluR1-siRNA in AC5 KO mice partially suppressed behavioral choices for AC5 KO pellets over WT ones (Figure 7C). These results indicate that the mGluR1 signaling pathway plays an essential role in the production of preferred food-choice behaviors.

The administration of LY341495 (mGluR2/3 antagonist) in WT mice induced behavioral preferences for AC5 KO pellets, mimicking the phenotype of AC5 KO mice (Figure 7D). Conversely, the administration of LY354740 (mGluR2/3 agonist) in WT mice with cut whiskers blocked the preferred choice for AC5 KO pellets to the level that WT mice with normal whiskers displayed (Figure 7E). These results suggest a role for mGluR2/3 in behavioral preferences for AC5 KO pellets. However, mGluR2 KO mice showed no preference for AC5 KO pellets over WT ones and for rough pellets over smooth ones (Figure 7F and G). mGluR2 KO mice with cut whiskers preferred AC5 KO pellets (Figure 7H). Thus, mGluR2 KO mice behaved as if WT mice. The stereotaxic injection of mGluR3-siRNA in the dorsal striatum of WT mice induced behavioral preferences for AC5 KO pellets and for rough pellets (Figure 7I and J). Thus, inhibition of mGluR3 in the dorsal striatum mimicked AC5 KO phenotypes.

The functional significance of CaMKIIα signaling activated by whisker trimming in WT mice and in AC5 KO mice (Figure 6D) was examined. The siRNA-mediated suppression of CaMKIIα in the dorsal striatum in WT mice with cut whiskers blocked preferred choices for AC5 KO pellets. Similarly, injection with CaMKIIα-siRNA in the dorsal striatum in AC5 KO mice suppressed preferred choices for AC5 KO pellets (Figure 7K and L).

## Discussion

Goal-directed actions are learned behaviors that depend on expected outcome values [5]. Habits initially form by prior experiences, although once formed, they are automatically expressed on the basis of acquired action values [2,37]. In contrast to these learned behaviors, behavioral preferences for the olfactory cue-directed option shown by AC5 KO mice or WT mice with cut whiskers were not formed through prior experience, but driven by programmed action values. These behavioral preferences occurred in a moderately compulsive manner over the presence of quinine (Figure 3). Nonetheless, these behaviors were predictably modified by increasing the activity of the corticostriatal input through whisker trimming or activation or activation/inhibition of mGluR receptors systems and AC5 in the dorsal striatum (Figures 6 and 7).

Habits and innate behaviors are expressed in the absence of conscious selection. This type of behaviors is resistant to modification by learning [1,3]. Certain forms of behavioral decisions in drug addiction, obsessive compulsive disorder (OCD), schizophrenia, and autism are also resistant to modification by learning learning [4,5]. Recent studies show that corticostriatal pathways represent the neural correlates involved in altered behavioral decisions in drug addiction [38], OCD [39,40], schizophrenia [41], and autism [42]. These results suggest that changing the neuronal activity in the corticostriatal pathways through an appropriate means can modify rigid behaviors. In fact, repeated optogenetic stimulation of the orbitofrontal cortex (OFC)–ventromedial striatum (VMS) generates OCD-like behavior [43], and optogenetic stimulation of lateral orbitofronto-striatal pathway suppresses repetitive, compulsive behaviors displayed by mice deficient for the synaptic scaffolding gene, Sapap3 [44]. The current study presents a novel opportunity to model innate-like behavior and its modification based on molecular mechanisms. AC5 KO mice or WT mice with trimmed whiskers may be used as an animal model to study the role of the corticostriatal circuit regulating innate-like or related behaviors.

**Figure 8:**
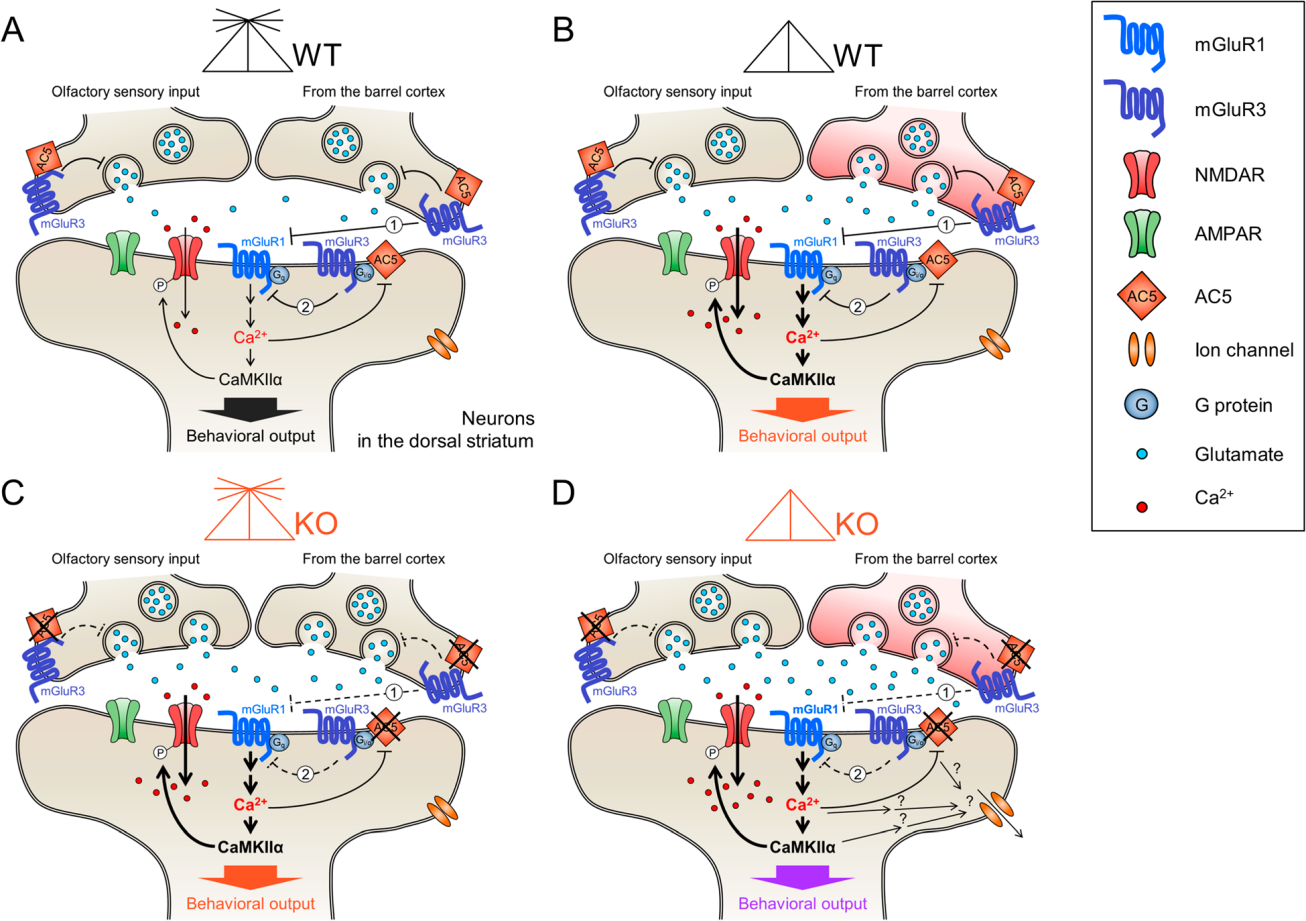
**A working hypothesis for the generation of behavioral preferences for olfactory- and tactile-cue-directed options. (A, B)** Olfactory and whisker sensory pathways in WT mice project to the dorsal striatum, which contains mGluR1, mGluR3, NMDA receptors, AC5, and CaMKIIα as key components of the decision making system **(A)**. Whisker trimming in WT mice **(B)** increases the corticostriatal synaptic input from the barrel cortex, which activates mGluR1 and its down-stream signaling cascade including CaMKIIα. Consequently whisker trimming modulates behavioral outputs in choosing olfactory- and tactile-cue-directed options. **(C, D)** The AC5 deficiency disrupts the mGluR3-mediated suppression of mGluR1, resulting in the activation of mGluR1 and a down-stream signaling cascade including CaMKIIα, and consequently behavioral outputs **(C)**. Whisker trimming in AC5 KO mice **(D)** increases the corticostriatal synaptic input from the barrel cortex, which activates mGluR1 and a down-stream signaling cascade including CaMKIIα; whereas the AC5 KO lead to the activation of mGluR1 and a down-stream signaling cascade. It is possible that whisker trimming in AC5 KO mice activates secondary signaling systems in the dorsal striatum, which are not switched on singly by AC5 KO or whisker trimming alone. Although it is unknown whether mGluR3 is colocalized with mGluR1 in the same post-synaptic neurons, whether mGluR3 and mGluR1 are located in different cells in the dorsal striatum, or whether mGluR3 is located in the presynaptic terminals of corticostriatal inputs, this model envisions that mGluR3 antagonistically suppresses the function of mGluR1 (pathways ① and ②).

Behavioral preferences for cue-oriented options displayed by AC5 KO mice have a feature of programmed ones. However, these behaviors were modified by changing the activity of the corticostriatal input or the neural activity in the dorsal striatum. Even whisker trimming in WT mice induced these behaviors. The effect of whisker trimming on the production of preferred food choices in WT mice appear to be mediated by the enhanced activation of mGluR1 and its downstream signaling cascade, including an increase in p-CaMKIIα level (Figure 6D, 7B, and 7K). Whereas the AC5 knockout effect on the food-choice behavior is likely produced by blocking of mGluR3 and its downstream signaling cascade in the dorsal striatum (Figure 7I, 7J and 7L). We speculate that the gain and loss of behavioral preferences for a specific cue-directed option are determined by cellular factors in the dorsal striatum, such that the preferred food choices are switched on when either the mGluR3-AC5 pathway is inactive or the mGluR1 pathway is active, whereas the preferred food-choices are switched off when mGluR1 or its downstream pathway is suppressed. These molecular switches are illustrated in Figure 8. In this model, we summarize that AC5 and mGluRs function as molecular switches to direct decisions on preferred food-choice behaviors. After whisker trimming in AC5 KO mice, the level of p-CaMKIIα in the dorsal striatum did not return to WT level (Figure 6D), although their behaviors were reverted (Figure 4F and G). These results suggest that AC5 KO mice with cut whiskers are not simple revertants to WT mice, but to something third ones (Figure 8), although their behavioral outputs for food-choices were similar to WT mice.

Whisker trimming increased the glutamatergic input from the barrel cortex into the dorsal striatum. Several lines of evidence to support the functional connectivity between whisker sensory inputs and the dorsal striatum. First, whisker sensory information is projected into the barrel cortex [32,45]. Second, we showed that whisker trimming induced c-Fos expression (Figure 6B) and p-CAMKIIα (Figure 6D) in the dorsal striatum. Third, injection of PTX into the barrel cortex increased c-Fos expression (Figure 6C) and p-CAMKIIα (Figure 6E) in the dorsal striatum. As described, GABAergic interneurons regulate the excitatory tone of glutamatergic projection neurons [33]. Fourth, after injecting a retrogracer (biotinylated dextran amine) into the dorsal striatum, neurons in the barrel cortex were labeled [46]. Fifth, injection of an AAV vector carrying eGFP in the barrel cortex labeled the axonal projection of neurons from the barrel cortex into the dorsal striatum (Figure 6A). Finally, injection of siRNA-mGluR1 (Figure 6B) or siRNA-CaMKIIα (Figure 6K) blocked whisker trimming effects in the preference for KO food. Together, these results support that the barrel cortex maintains a direct and functional connectivity with the dorsal striatum, which represents the neural mechanism as to how AC5 KO mice produced preferred food-choice for KO pellets. Considering that whisker trimming should induce physiological levels of changes in the dorsal striatum, whisker trimming method may be used as a tool for studies of various striatal functions.

Whisker trimming in WT mice drove animals to choose food pellets with a specific olfactory cue, but it did not drive to choose food pellets with a tactile cue (rough pellets), whereas AC5 KO mice expressed both choice phenotypes. Because the injection of AC5-siRNA in the striatum of WT mice replicated the production of preferred choices for AC5 KO food pellets and rough pellets (Figure 5), we predict the presence of the corticostriatal loop that has a modulatory role in the behavioral choice of rough pellets, but is distinguished from that for KO food pellets. Regarding that siRNA-mediated suppression of mGluR3 in the dorsal striatum (Figure 7I and J) mimicked behavioral phenotypes of AC5 knockdown effects (Figure 5), AC5 likely functions as a mediator for mGluR3, as it is for D2 dopamine receptors [23] and MOR and DOR opioid receptors [25]. Considering that the whisker sensory input pathway modulated the behavioral choice of AC5 KO food through mGluR1 (Figure 7B), it is possible that other unidentified synaptic input that impinges upon AC5-coupled receptors in the dorsal striatum regulates the preferred choice for rough pellets.

Stereotaxic injection of siRNA for a gene of interest in the brain can be used to investigate the role of a target gene in the production of behaviors, as demonstrated previously [29] and in the present study. As demonstrated in the present study, stereotaxic injection of siRNA for AC5, mGluR1, mGluR3, and CaMKIIα in the dorsal striatum and following behavioral assessments made it possible elaborate the role of each gene in the production of behavioral preferences for specific-cue-directed foods. Furthermore, this is a powerful tool to dissect the role of specific genes in specific brain regions in animals.

Most mammals including all primates, except human, develop vibrissae on the face [47]. Given that the barrel cortex undergoes highly plastic changes after whiskers are trimmed [48] and whisker trimming in mice increases the corticostriatal synaptic input (Figure 6), the evolutionary loss of whiskers in humans may be projected into the relevant modification, such as the tonic enhancement of the sensory motor-corticostriatal synaptic input. AC5 KO mice did not show altered preference for urine, cage-bed and blood serum (Figure 2G-I). Therefore, we doubt that preferred food-choice for KO food displayed by AC5 KO mice is attributed to general increase of vigilance in the olfactory perception system. Whereas AC5 KO mice and WT with cut whiskers showed an enhanced sensitivity to quinine (Figure 3D and E). We do not exclude the possibility that whisker trimming or knocking down of AC5 increases a specific sensitivity to certain odor or taste cues. Although it might be too speculative yet, it will be interesting to know whether humans have had an opportunity to gain a novel behavioral trait in the food-intake pattern based on their keen sensory discrimination ability at the expense of a whisker system. Further studies should be necessary.

## Conclusions

The present study describes the molecular mechanism underlying behavioral preferences in specific cue-oriented food-intakes. AC5 KO mice consumed food pellets nearly one after another in their cages. The unusual behaviors displayed by AC5 KO mice were due to the gain of a novel behavioral trait of behavioral preferences for food pellets that had been taking, which wild-type mice normally ignored. Behavioral preferences for AC5 KO pellets in AC5 KO mice occurred compulsively in the presence of quinine. These food-choice behaviors in AC5 KO mice disappeared after whisker trimming, whereas whisker trimming in wild-type mice oppositely induced the same preferred food-choice behaviors for AC5 KO-food pellets as AC5 KO mice displayed. Our results show that the cortico-striatal input and the dorsal striatum which contains mGluR1, mGluR3, AC5, and CaMKIIα regulate these food-choice behaviors. As being contrasting to learned behaviors, behavioral preferences for the olfactory cue-directed option shown by AC5 KO mice or WT mice with cut whiskers were not formed through prior experience, but occurred through intrinsic action values. The gain and loss of behavioral preferences for a specific cue-directed option were produced by changing the activity of specific cellular factors in the dorsal striatum, such that the preferred food choices were switched on when either the mGluR3-AC5 pathway was inactive or the mGluR1 pathway was active, whereas the preferred food-choices were switched off when mGluR1 or its downstream pathway was suppressed. These results identify the AC5 and mGluR system in the dorsal striatum as molecular on/off switches to direct decisions on behavioral preferences for cue-directed options.

## Methods

### Animals

AC5 KO mice were maintained as reported previously [23]. mGluR2 KO embryos [49] were obtained from RIKEN BioResource Center (Tsukuba Ibaraki, Japan) and transferred to foster mother mice to revive mGluR2 KO mice. Genotyping was carried out using the following primer sets: 5′-GTCCGAGGATGGAGGCAGTT-3′ and 5′-CACCACTGCAATGAGCGCATA-3′ for AC5 WT (373 bp); 5′-ACCGTCGAGGATGGAGACGG-3′ and 5′-TGTCCATCTGCTGCACGAGACTA-3′ for AC5 KO (514 bp); 5′-CTTAGGTTCCTGGCACTGCT-3′, 5′-TTGATGCGGTCCAGTGCAA-3′ and 5′-AACTGTTCGCCAGGCTCAA-3′ for mGluR2 WT (502 bp) and mGluR2 KO (200 bp). Mice were housed in a temperature- and humidity-controlled environment with a 12 h light/dark cycle and were allowed access to food and water *ad libitum*. All experiments were conducted in accordance with the Guidelines of Animal Care at Ewha Womans University through permission of EWU- IACUC (No. 2013-02-008).

### Preparation of WT pellets, KO pellets, food pellet-surface eluate, and quinine-pasted pellets

Food pellets collected from cages containing WT mice were termed WT pellets and those from cages containing AC5 KO mice, KO pellets. Rough food pellets were prepared by carving the surface of regular fresh food pellets with a knife to have 5 longitudinal grooves (3 mm in width, 10 mm in spacing, 4 mm in depth), while smooth pellets were regular fresh food pellets obtained from a food supplier. Each individual was placed in a normal cage presented with food pellets and the amounts of food pellets remaining the next day were recorded. The surface (<1 mm in depth) of food pellets was collected, and incubated in 4 volumes of water (weight (g): vol (ml) = 1:4) at 25°C with shaking at 120 rpm for 20 min. The supernatant containing the eluate of fresh, WT pellets or KO pellets were separately collected after centrifugation at 1,000 rpm for 3 min. Fresh food pellets were brushed with 0.1 ml of the indicated eluate, and the amount of food intake by a mouse in a day was recorded. Quinine at the indicated concentration was pasted to the surfaces of food pellets using a brush as above.

### Real time PCR analysis

Real-time PCR analysis was carried out as described previously [50], using the CFX 96 Real-Time PCR System Detector (Bio-Rad Laboratories; Foster City, CA, USA) and the following primer sets: 5′-GGGAGAACCAGCAACAGG-3′ and 5′- CATCTCCATGGCAACATGAC-3′ for AC5; 5′-GCTGCCATCTGTTTTACGG-3′and 5′- TGACTGGTGCCTGATGAACT-3′ for GAPDH; 5′-GCTGCCATCTGTTTTACGG-3′ and 5′-TGACTGGTGCCTGATGAACT-3′, AC5 primer set; 5′-GGGAGAACCAGCAACAGG-3′ and 5′-CATCTCCATGGCAACATGAC −3′ for L32.

### Stereotaxic injection of siRNA, Lenti-shRNA-AC5, and AAV-CaMKIIa-eGFP

Stereotaxic injection of siRNA was performed as described [29]. In brief, mice were anesthetized with the mixture (3.5: 1) of ketamine hydrochloride (50 mg/ml) and xylazine hydrochloride (23.3 mg/ml) at a dose of 2.5 μl/g body weight. siRNA-control (SN-1012), mGluR1-siRNA (1065624, NM_000838.2), mGluR3-siRNA (1366186, NM181850.1) and NR2B-siRNA (1366085, NM_008171.2) were purchased from Bioneer Co. (Deajun, Korea). The FAM-labeled RISK-independent siRNA transfection control siGLO Green (D-001630-01-05), AC5-siRNA and CaMKIIα-siRNA (M-059173–00–0005, NM_009792) were purchased from Dharmacon Inc. (Chicago, IL, USA). One volume of the diluted (50 ng/μl) siRNA-control, siRNA-target gene, after adding siGLO Green (19:1 ratio), was mixed with 2.5 volume of neurofect transfection reagent (T800075; Genlantis, San Diego, CA, USA) and 0.5 volume of 50% sucrose. The siRNA-target gene was injected in a volume of 1.5 μl (18 ng siRNA) into each dorsal striatum (AP, +1.0; ML, ±1.5; DV, −3.6 mm). Behavioral tests were performed 24 h after the siRNA injection. Injection sites were histologically examined for fluorescence from siGLO Green, and mice with mislocalized injections were excluded from the final data.

Lenti-shRNA-AC5 was purchased from Open Biosystem (RMM3981-97078870; Open Biosystem, USA), and was packed and amplified to 10^12^ pfu with the help of Macrogen (Seoul, Republic of Korea). Lenti-shRNA-AC5 was injected into the each dorsal striatum (stereotaxic coordinate: AP, +1.0; ML, ±1.5; DV, −3.6 mm) or nucleus accumbens (AP, +1.0; ML, ±1.5; DV, −4.9 mm) in a volume of 1.5 μl containing 10^9^ pfu using a Hamilton syringe with a 30-gauge needle. Injected mice were maintained in normal home cages in pairs. Ten days after the injection, behavioral tests were performed.

AAV-CaMKIIa-eGFP(AV-1-PV1917) was purchased from Penn Vector Core (Upenn, Philadelphia, PA, USA) and injected into the barrel cortex (AP, −0.9; ML, −3.0; DV, −1.7 mm) in a volume of 1 μl carrying 3.65 × 10^9^ viral particles/μl. The GFP signal was detected 10 days after injection using a fluorescent microscope.

### Infusion of siRNA-Gαolf into the olfactory epithelium

Mice were anesthetized with ketamine and xylazine, and placed on a pad warmed to 37°C. The siRNA-Gαolf mixture containing 2 volumes of 270 ng/μl (20 μM) siRNA-Gαolf (14680, Bioneer, Korea), 1 volume of 50% sucrose and 5 volumes of oligofectamine (12252–011, Invitrogen, USA) was dropped into the olfactory epithelium through each nostril, each with a volume of 2 μl at 5-min intervals for a total of 4 times. Food choice test was performed between 48 h and 60 h after the infusion. Gαolf expression levels in the olfactory epithelium were examined after 24, 48 and 72 h of siRNA infusion.

### Drug administration

Animals were individually anesthetized with ketamine and xylazine during the implantation of a 26-gauge guide cannula (C315G/SPC, Plastics One, Bilaney, UK). The guide cannula was secured by a dummy cannula (C313DC, Plastics One). Drugs were infused into the dorsal striatum through a 33-gauge internal cannula (C315I, Plastics One) inserted into the guide cannula while anesthesizing with 1.2% isoflurane (99% oxygen) [25]. 3,5-dihydroxyphenylglycine (DHPG; 0342, Tocris) was infused directly into the dorsal striatum (AP, +1.0; ML, ±1.5; and DV, −3.6 mm) through the pre-implanted cannulas at the dose of 20 nmol/injection. Picrotoxin (1.5 nmol/injection) was delivered in a volume of 1.5 μl by a stereotaxic injection into the left side of the barrel cortex (AP, −0.9; ML, −3.0; and DV, −1.7 mm). DHPG and picrotoxin were delivered while animals were individually anesthetized with 1.2% isoflurane. LY341495 and LY354740 were intraperitoneally administered at the dose of 1 mg/kg.

### Western blot analysis and immunohistochemistry

Western blot analysis and immunohistochemical analyses were carried out as described previously [25,27]. Polyclonal anti-Gαolf (1:1,000, sc-385, Santa Cruz Biotech, CA, USA), monoclonal anti-CaMKIIα (1:3,000; sc-13141; Santa Cruz Biotech) and polyclonal anti-phospho-CaMKIIα (1:1,000; sc-12886; Santa Cruz Biotech) were used. Enhanced chemiluminescence (ECL) system (EBP-10073; Elpis Biotech, Daejeon, Korea) was used for visualization and quantification was done using Image J (NIH, USA).

The brain was sectioned by a vibratome (Leica VT 1000S; Leica Instruments, Nussloch, Germany) at a 40-μm thickness. The levels of c-Fos-positive cells were evaluated using the Olympus BX51 microscope equipped with DP71 camera and the METAMorph Microscopy Automation & Image Analysis software (Molecular Devices, Sunnyvale, CA, USA).

### Statistical analysis

Two-sample comparisons were conducted using the two-tailed t-test, and multiple comparisons were made using one-way, two-way, or two-way repeated-measures ANOVA followed by a *post hoc* test using Graphpad Prism 6 (San Diego, CA, USA) and SPSS 19 (IBM, New York, NY, USA). All data are presented as the mean ± SEM, and a statistical difference was accepted at the 5% level.

## References

[1] Dolan RJ, Dayan P (2013). Goals and habits in the brain. Neuron.

[2] Hilario MR, Costa RM (2008). High on habits. Front Neurosci.

[3] Tierney AJ (1986). The evolution of learned and innate behavior: contributions from genetics and neurobiology to a theory of behavioral evolution. Anim Learn Behav.

[4] Murphy DL, Timpano KR, Wheaton MG, Greenberg BD, Miguel EC (2010). Obsessive-compulsive disorder and its related disorders: a reappraisal of obsessive-compulsive spectrum concepts. Dialogues Clin Neurosci.

[5] Lee D (2013). Decision making: from neuroscience to psychiatry. Neuron.

[6] Wunderlich L, Dayan P, Dolan RJ (2012). Mapping value based planning and extensively trained choice in the human brain. Nat Neurosci.

[7] Sjoerds Z, de Wit S, van den Brink W, Robbins TW, Beekman AT, Penninx BW, Veltman DJ (2013). Behavioral and neuroimaging evidence for overreliance on habit learning in alcohol-dependent patients. Transl Psychiatry.

[8] Fernandez-Ruiz J, Wang J, Aigner TG, Mishkin M (2001). Visual habit formation in monkeys with neurotoxic lesions of the ventrocaudal neostriatum. Proc Natl Acad Sci U S A.

[9] Cai X, Kim S, Lee D (2011). Heterogeneous coding of temporally discounted values in the dorsal and ventral striatum during intertemporal choice. Neuron.

[10] Sul JH, Kim H, Huh N, Lee D, Jung MW (2010). Distinct roles of rodent orbitofrontal and medial prefrontal cortex in decision making. Neuron.

[11] Quinn JJ, Pittenger C, Lee AS, Pierson JL, Taylor JR (2013). Striatum-dependent habits are insensitive to both increases and decreases in reinforcer value in mice. Eur J Neurosci.

[12] Dunnett SB, White A (2006). Striatal grafts alleviate bilateral striatal lesion deficits in operant delayed alternation in the rat. Exp Neurol.

[13] Pauli WM, Clark AD, Guenther HJ, O’Reilly RC, Rudy JW (2012). Inhibiting PKMζ reveals dorsal lateral and dorsal medial striatum store the different memories needed to support adaptive behavior. Learn Mem.

[14] Vertes RP (2004). Differential projections of the infralimbic and prelimbic cortex in the rat. Synapse.

[15] Sousa N, Almeida OF (2012). Disconnection and reconnection: the morphological basis of (mal) adaptation to stress. Trends Neurosci.

[16] Doya K (2008). Modulator of decision making. Nat Neurosci.

[17] Fidalgo C, Conejo NM, González-Pardo H, Arias JL (2011). Cortico-limbic-striatal contribution after response and reversal learning: a metabolic mapping study. Brain Res.

[18] Yin HH, Knowlton BJ, Balleine BW (2004). Lesions of dorsolateral striatum preserve outcome expectancy but disrupt habit formation in instrumental learning. Eur J Neurosci.

[19] Ashby FG, Turner BO, Horvitz JC (2010). Cortical and basal ganglia contributions to habit learning and automaticity. Trends Cogn Sci.

[20] Middleton FA, Strick PL (2000). Basal ganglia output and cognition: evidence from anatomical, behavioral, and clinical studies. Brain Cogn.

[21] Voorn P, Vanderschuren LJ, Groenewegen HJ, Robbins TW, Pennartz CM (2004). Putting a spin on the dorsal-ventral divide of the striatum. Trends Neurosci.

[22] Juri C, Rodriguez-Oroz M, Obeso JA (2010). The pathophysiological basis of sensory disturbances in Parkinson’s disease. J Neurol Sci.

[23] Lee KW, Hong JH, Choi IY, Che Y, Lee JK, Yang SD, Song CW, Kang HS, Lee JH, Noh JS, Shin HS, Han PL (2002). Impaired D2 dopamine receptor function in mice lacking type 5 adenylyl cyclase. J Neurosci.

[24] Matsuoka I, Suzuki Y, Defer N, Nakanishi H, Hanoune J (1997). Differential expression of type I, II, and V adenylyl cyclase gene in the postnatal developing rat brain. J Neurochem.

[25] Kim KS, Lee KW, Lee KW, Im JY, Yoo JY, Kim SW, Lee JK, Nestler EJ, Han PL (2006). Adenylyl cyclase type 5 (AC5) is an essential mediator of morphine action. Proc Natl Acad Sci U S A.

[26] Kim KS, Kim H, Baek IS, Lee KW, Han PL (2011). Mice lacking adenylyl cyclase type 5 (AC5) show increased ethanol consumption and reduced ethanol sensitivity. Psychopharmacology (Berl).

[27] Kim KS, Kim H, Park SK, Han PL (2012). The dorsal striatum expressing adenylyl cyclase-5 controls behavioral sensitivity of the righting reflex to high-dose ethanol. Brain Res.

[28] Park HY, Kang YM, Kang Y, Park TS, Ryu YK, Hwang JH, Kim YH, Chung BH, Nam KH, Kim MR, Lee CH, Han PL, Kim KS (2014). Inhibition of adenylyl cyclase type 5 prevents L-DOPA-induced dyskinesia in an animal model of Parkinson’s disease. J Neurosci.

[29] Kim KS, Lee KW, Baek IS, Lim CM, Krishnan V, Lee JK, Nestler EJ, Han PL (2008). Adenylyl cyclase-5 activity in the nucleus accumbens regulates anxiety-related behavior. J Neurochem.

[30] Kim KS, Kim J, Back SK, Im JY, Na HS, Han PL (2007). Markedly attenuated acute and chronic pain responses in mice lacking adenylyl cyclase-5. Genes Brain Behav.

[31] Kim KS, Han PL (2009). Mice lacking adenylyl cyclase-5 cope badly with repeated restraint stress. J Neurosci Res.

[32] Petersen CC, Sakmann B (2000). The excitatory neuronal network of rat layer 4 barrel cortex. J Neurosci.

[33] Zhang G, Gao Z, Guan S, Zhu Y, Wang JH (2013). Upregulation of excitatory neurons and downregulation of inhibitory neurons in barrel cortex are associated with loss of whisker inputs. Mol Brain.

[34] Berretta S, Parthasarathy HP, Graybiel AM (1997). Local release of GABAergic inhibition in the motor cortex induces immediate-early gene expression in indirect pathway neurons of the striatum. J Neurosci.

[35] Schoepp DD (2001). Unveiling the functions of presynaptic metabotropic glutamate receptors in the central nervous system. J Pharmacol Exp Ther.

[36] Sharko AC, Hodge CW (2008). Differential modulation of ethanol-induced sedation and hypnosis by metabotropic glutamate receptor antagonists in C57BL/6 J mice. Alcohol Clin Exp Res.

[37] Dezfouli A, Balleine BW (2013). Actions, action sequences and habits: evidence that goal-directed and habitual action control are hierarchically organized. PLoS Comput Biol.

[38] Hyman SE, Malenka RC, Nestler EJ (2006). Neural mechanisms of addiction: the role of reward-related learning and memory. Annu Rev Neurosci.

[39] Stein DJ (2000). Neurobiology of the obsessive-compulsive spectrum disorders. Biol Psychiatry.

[40] Friedlander L, Desrocher M (2006). Neuroimaging studies of obsessive-compulsive disorder in adults and children. Clin Psychol Rev.

[41] Sorg C, Manoliu A, Neufang S, Myers N, Peters H, Schwerthöffer D, Scherr M, Mühla M, Zimmer C, Drzezga A, Förstl H, Bäuml J, Eichele T, Wohlschläger AM, Riedl V (2013). Increased intrinsic brain activity in the striatum reflects symptom dimensions in schizophrenia. Schizophr Bull.

[42] Langen M, Leemans A, Johnston P, Ecker C, Daly E, Murphy CM, Dell’acqua F, Durston S, Consortium AIMS, Murphy DG (2012). Fronto-striatal circuitry and inhibitory control in autism: findings from diffusion tensor imaging tractography. Cortex.

[43] Ahmari SE, Spellman T, Douglass NL, Kheirbek MA, Simpson HB, Deisseroth K, Gordon JA, Hen R (2013). Repeated cortico-striatal stimulation generates persistent OCD-Like behavior. Science.

[44] Burguiere E, Monteiro P, Feng G, Graybiel AM (2013). Optogenetic stimulation of lateral orbitofronto-striatal pathway suppresses compulsive behaviors. Science.

[45] Aronoff R, Matyas F, Mateo C, Ciron C, Schneider B, Petersen CC (2010). Long-range connectivity of mouse primary somatosensory barrel cortex. Eur J Neurosci.

[46] Alloway KD, Lou L, Nwabueze-Ogbo F, Chakrabarti S (2006). Topography of cortical projections to the dorsolateral neostriatum in rats: multiple overlapping sensorimotor pathways. J Comp Neurol.

[47] Van Horn RN (1970). Vibrissae structure in the Rhesus monkey. Folia Primatol.

[48] Durham D, Woolsey TA (1978). Acute whisker removal reduces neuronal activity in barrels of mouse SmL cortex. J Comp Neurol.

[49] Yokoi M, Kobayashi K, Manabe T, Takahashi T, Sakaguchi I, Katsuura G, Shigemoto R, Ohishi H, Nomura S, Nakamura K, Nakao K, Katsuki M, Nakanishi S (1996). Impairment of hippocampal mossy fiber LTD in mice lacking mGluR2. Science.

[50] Seo JS, Park JY, Choi J, Kim TK, Shin JH, Lee JK, Han PL (2012). NADPH oxidase mediates depressive behavior induced by chronic stress in mice. J Neurosci.

